# Somatotopic localization of c-Fos expression in the spinal cord in response to noxious heat sensation

**DOI:** 10.3389/fnana.2022.1035257

**Published:** 2022-09-28

**Authors:** Safa Shehab, Hayate Javed, Gulfaraz Khan

**Affiliations:** ^1^Department of Anatomy, College of Medicine and Health Sciences, United Arab Emirates University, Al Ain, United Arab Emirates; ^2^Department of Medical Microbiology and Immunology, College of Medicine and Health Sciences, United Arab Emirates University, Al Ain, United Arab Emirates

**Keywords:** c-Fos, heat sensation, spinal cord, resiniferatoxin, transient receptor potential vanilloid type 1 (TRPV1)

## Introduction

**Figure 1:**
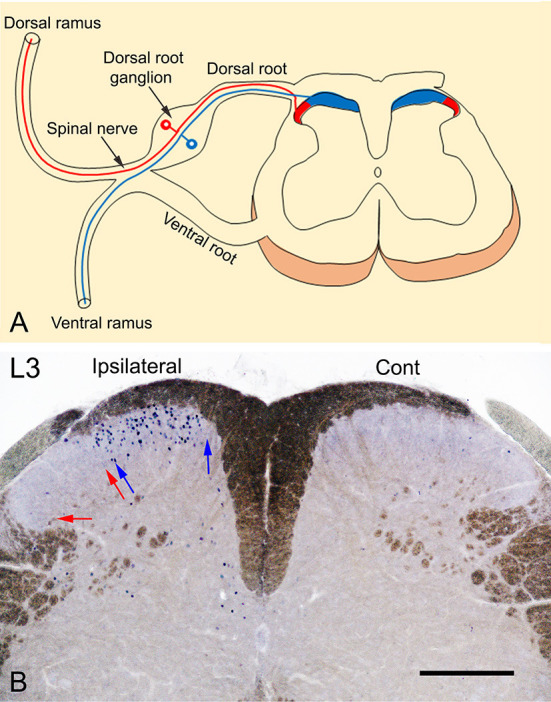
**(A)** Somatotopic arrangement of the termination of primary afferent fibers. Primary afferents of the dorsal ramus terminate in the lateral 1/3 (red) while those from the ventral ramus, terminate in the medial 2/3 (blue) of the superficial layer of the dorsal horn of the spinal cord. **(B)** A transverse section of L3 spinal segment showing the neuronal expression of c-Fos in response to noxious heat stimulation by immersion of the left foot in 52°C hot water bath. The photography was intentionally carried out without dehydration of the sections to reveal the myelinated fibers (brown color) and to show a clear demarcation of the dorsal horn of the spinal cord. The expression of c-Fos is observed only in the medial 1/2 of the superficial layers (laminae I-II) of the ipsilateral dorsal horn marked between blue arrows where the primary afferents of the foot terminate. In comparison, no c-Fos expression could be observed in the lateral 1/2 of the dorsal horn marked between red arrows where the primary afferents of the back on the body terminate or in the contralateral control side. Scale bar = 250 μm.

We have read with great interest the manuscript entitled “A novel spinal neuron connection for heat sensation” by Wang et al. (2022) published in Neuron 110, 1–19, 2022. In this study the authors used c-Fos::shEGFP mice that express a short half-life enhanced green fluorescent protein (shEGFP) under the control of endogenous c-Fos promoter to characterize the neurons which are involved in heat sensations. When mice were exposed to noxious heat stimuli by using 52°C hot plate, compared with mice exposed to 25°C plate, shEGFP expression was increased in neurons of spinal cord dorsal horns (Figures 1B,C, in Wang et al., 2022). The authors confirmed that those shEGFP +ve neurons also overlapped with the same neurons labeled with c-Fos antibody, demonstrating its validity as a good indicator of activated neurons in the dorsal horn of the spinal cord. shEGFP +ve and c-Fos +ve neurons were found mainly in the superficial layer of the dorsal horn of the spinal cord where nociceptive primary afferents (mainly C and Aδ fibers) normally terminate. The shEGFP+ neurons were individually isolated and subjected to single-cell RT-PCR to determine the co-expression of a number of other neuronal markers. Results showed that 78% of shEGFP +ve cells were positive for excitatory neurons which contain Vglut2 (vesicular glutamate transporter) whereas 22% were positive for inhibitory neurons which contain GAD65/67 (glutamate decarboxylase 65/67) (Figures 1G,H, in Wang et al., 2022). Moreover, following noxious heat, 33% of c-Fos +ve neurons also expressed ErbB4 (Figures 1M,N, in Wang et al., 2022). In addition to several other techniques used in this study, the authors concluded that the data provide evidence for the involvement of spinal ErbB4 +ve neurons in heat hypersensitivity in inflammatory and neuropathic pain.

Our main concern is with the distribution of the shEGFP +ve and c-Fos +ve neurons in this study. In their Figure 1, the authors showed the distribution of c-Fos immunolabeled neurons extending mediolaterally in the superficial layer of the dorsal horn of the spinal cord in response to heat application to the hind paw. In this kind of treatment, one should expect to observe c-Fos labeled neurons only in the medial portion of the dorsal horn.

## Somatotopic termination of the primary afferent fibers

The spinal peripheral nerves are formed by the union of the dorsal and ventral roots which then divide into dorsal and ventral rami. The dorsal ramus supplies the posterior aspect of the body and the skin of the back while the ventral ramus supplies the anterior aspect of the body and the limbs. The terminations of the primary afferents of these rami are somatotopically arranged in the spinal cord (Molander and Grant, 1986; Grant, 1993; Takahashi et al., 2002; Shehab et al., 2008; Shehab, 2009; Shehab and Hughes, 2011). The medial part of the dorsal horn receives the termination of the primary afferents from the limbs through the ventral ramus, while the lateral part receives the termination of the primary afferents from the skin of the back through the dorsal ramus (Figure 1A). Therefore, exposing the hind paw of mice to noxious heat stimuli, as carried out by Wang et al. (2022), should cause neuronal activation which in turn would lead to c-Fos induction in the medial part of the dorsal horn. This has been extensively demonstrated in rodents in different laboratories in which c-Fos was induced by either radiant heat pulses (52°C; Figures 2, 3 in Hunt et al., 1987) or the injection of formalin into plantar skin (Figures 1, 3 in Todd et al., 1994) or the immersion hind paw in 55°C water bath (Figure 4E in Cavanaugh et al., 2009).

## Localization of c-Fos expression in response to noxious heat stimulation of the hind paw

We used two methods to localize the distribution of c-Fos expression in response to noxious heat stimulation in adult male mice (C57BL/6): (1) Intraplantar injection of resiniferatoxin (45 μl of 0.001%), as an agonist of heat receptor TRPV1 (transient receptor potential vanilloid type 1, Caterina et al., 2000) into the left hind paw. (2) Immersion of the hind paw in 52°C hot water bath (20 s followed by 10 s off and then 20 s in). Both of these treatments were carried out under isoflurane (2–2.5%) inhalational anesthesia. Ninety minutes after the treatment, the animals were sacrificed by perfusing with 4% paraformaldehyde. All experimental procedures were approved by the Animal Ethics Committee of the CMHS, UAE University, and were performed in accordance with the guidelines of the European Communities Council Directive of November 24, 1986 (86/609/EEC). Cryostat sections (50 μm thickness) of lumbar spinal cord segments were prepared and stained immunohistochemically with c-Fos antibody (1:5000; Merck Millipore, Cat No. ABE457; Lot# 3728278) as previously described (Todd et al., 1994). Figure 1B shows c-Fos immunolabeled neurons located only in the medial part of the superficial layers (laminae I-II) of the spinal cord compared with the absence of labeling in contralateral control side. These findings and those from previous studies (Hunt et al., 1987; Todd et al., 1994; Cavanaugh et al., 2009), indicate that the neuronal expression of c-Fos in the lateral part of the dorsal horn of the spinal cord reported by Wang et al. (2022) is probably non-specific and not induced in response to noxious heat stimuli applied to the plantar skin by the exposure to 52°C hot plate.

## Discussion and conclusion

Previous studies (Hunt et al., 1987; Todd et al., 1994; Cavanaugh et al., 2009) and our current results presented here, cast doubt on the validity of: (1) the expression of c-Fos in the spinal neurons as being activated by noxious heat stimuli, and (2) the characterization of c-Fos labeled neurons in the study of Wang et al. (2022).

## Author contributions

SS designed the article and planned the research. SS and HJ carried out the experiments. SS, HJ, and GK wrote, edited, and revised the manuscript. All authors contributed to the article and approved the submitted version.

## Funding

This work was supported by research grants from the UAE University (#12M095; 12M100; and 31R259).

## Conflict of interest

The authors declare that the research was conducted in the absence of any commercial or financial relationships that could be construed as a potential conflict of interest.

## Publisher's note

All claims expressed in this article are solely those of the authors and do not necessarily represent those of their affiliated organizations, or those of the publisher, the editors and the reviewers. Any product that may be evaluated in this article, or claim that may be made by its manufacturer, is not guaranteed or endorsed by the publisher.
